# A Delphi yarn: applying Indigenous knowledges to enhance the cultural utility of SMART Recovery Australia

**DOI:** 10.1186/s13722-020-00212-8

**Published:** 2021-01-06

**Authors:** Elizabeth Dale, Katherine M. Conigrave, Peter J. Kelly, Rowena Ivers, Kathleen Clapham, K. S. Kylie Lee

**Affiliations:** 1 Illawarra Health and Medical Research Institute, Wollongong, NSW Australia; 2 School of Psychology, University of Wollongong, Wollongong, NSW 2500 Australia; 3 Royal Prince Alfred Hospital, Drug Health Services, Camperdown, NSW Australia; 4 Faculty of Medicine and Health, Discipline of Addiction Medicine, NHMRC Centre of Research Excellence in Indigenous Health and Alcohol, The University of Sydney, Camperdown, NSW Australia; 5 Gradute School of Medicine, The University of Wollongong, Wollongong, NSW Australia; 6 Illawarra Aboriginal Medical Service, Wollongong, NSW Australia; 7 Ngarruwan Ngadju First Peoples Health and Wellbeing Research Centre, Australian Health Services Research Institute, Faculty of Business, University of Wollongong, Wollongong, NSW Australia; 8 Centre for Alcohol Policy Research, La Trobe University, Bundoora, VIC Australia

**Keywords:** Mutual support group, Indigenous, Addiction, Substance use, SMART recovery

## Abstract

**Background:**

Mutual support groups are a popular treatment for substance use and other addictive behaviours. However, little is known about the cultural utility of these programmes for Indigenous peoples.

**Methods:**

A three-round Delphi study, utilising Indigenous research yarning methods was conducted to: (1) Obtain expert opinion regarding the cultural utility of an Indigenous SMART Recovery handbook; (2) Gain consensus on areas within the SMART Recovery programme that require cultural modification and; (3) Seek advice on how modifications could be implemented in future programme design and delivery. The panellists were 11 culturally, geographically, and professionally diverse Indigenous Australian health and wellbeing experts. A group consensus level of 80% was set prior to each survey round.

**Results:**

There was 100% participant retention across all three Delphi rounds. The panel reached consensus on five key programme modifications (composition of a separate facilitator and group member handbook; culturally appropriate language, terminology, and literacy level; culturally meaningful programme activities; supplementary storytelling resources; and customisation for diverse community contexts). The panel also developed a series of practical implementation strategies to guide SMART Recovery through a modification process.

**Conclusion:**

The findings highlight the importance of involving Indigenous peoples in the design, delivery and validation of mainstream mutual support programmes. Indigenous-led programme modifications could help improve accessibility and usefulness of mutual support groups for Indigenous peoples worldwide. This study is an example of how Indigenous research methods can be used alongside the Delphi technique. This approach demonstrated a way that Indigenous peoples from culturally and geographically diverse locations can participate in research anonymously, autonomously and without added burden on personal, community or professional obligations.

## Background

Mutual support group programmes are a popular treatment for problems arising from substance use and other behaviours of addiction (e.g. gambling) [1, 2]. Such groups offer non-clinical, community-based meetings that harness experiential knowledge and mobilise member-to-member social, emotional, and informational support [3]. Treatment offered by such programmes is free to attend and offered on an ongoing basis [4].

The most prevalent mutual support group programmes are the 12-step modalities (e.g., Alcoholics Anonymous (AA) Gamblers Anonymous (GA)) and SMART Recovery. Research shows that regular group attendance can help build personal insight [5], enhance problem-solving skills [6] and promote long-term abstinence [7]. However, a recent systematic review by Dale et al. found that few studies have examined the ‘cultural utility’ of these popular programmes for Indigenous peoples (defined as perceived suitability and helpfulness) [8].

Underpinned by western knowledge and empiricism, there are tenets of the 12-step programmes and SMART Recovery that appear counter-cultural for Aboriginal and Torres Strait Islander peoples (hereafter referred to as “Indigenous Australians”). For example, AA is built upon western religious ideologies [9] that differ from Indigenous Australians’ notions of spirit and spirituality [10]. SMART Recovery is centred on western psychological theories (i.e., cognitive behavioural therapy and motivational interviewing) [11–14] that have not undergone cultural validation to demonstrate their therapeutic benefits for Indigenous peoples [15–18].

A small group of studies show that Indigenous Australians and First Nations American and Canadian peoples have begun to informally embed their cultures in AA [19–22] and SMART Recovery [23]. This has included linguistic substitutions [24], replacing western religious practices for traditional ceremonies [25], and omitting programme components perceived as being inconsistent with an Indigenous worldview of health and wellness [23]. Of these studies, just one [23] provided detailed examples of how SMART Recovery could be adjusted to better suit Indigenous Australians (based on Indigenous facilitators’ and group members’ feedback). One key recommendation was the need for culturally appropriate programme materials.

In 2014, SMART Recovery Australia received a small, one-off non-government grant to modify their original programme handbook for Indigenous Australian facilitators and group members. The resulting handbook contains the same core programme tools and operational features as the mainstream resource but is supplemented with Indigenous Australian artwork and words (e.g., “yarndi” (cannabis)). The handbook was co-created with Indigenous Australian health professionals who, at the time, were completing SMART Recovery facilitator training (n = 5; of which n = 4, New South Wales; n = 1, Victoria). However, since then, this Indigenous Australian handbook has not been formally integrated into the SMART Recovery programme (personal communication with SMART Recovery Australia). Neither has it been reviewed by a broader group of Indigenous Australians.

Therefore, the aim of this study was to consult with Indigenous Australian health and wellbeing experts to: (1) Obtain expert opinion regarding the cultural utility of the SMART Recovery Aboriginal and Torres Strait Islander programme handbook; (2) Gain consensus on areas within the programme that require cultural modification; and (3) Seek advice on how modifications could be implemented in future programme design and delivery.

## Methods

### An Indigenous-lensed Delphi design

The Delphi technique [26] was used to coordinate an iterative Indigenous research topic yarn [27] with a culturally, geographically and professionally diverse panel of Indigenous Australian health and wellbeing experts. The Delphi technique uses a series of questionnaire rounds to solicit consensus opinions from a group of experts [28]. Indigenous research topic yarning is a relational and culturally acceptable method for obtaining Indigenous peoples perspectives on a research topic [29]. Yarning was used instead of traditional interviews to avoid a question–answer dialogue and to ensure participants’ cultural safety.

The Delphi technique was chosen over other consensus methods (e.g. focus groups) because it enabled our panellists to participate despite differing geographical locations, time zones and professional, community or personal obligations [30]. The anonymity, autonomy and relational nature of the Delphi technique [31] was also compatible with Indigenous research principles (relationality, reciprocity and respect) [32–34]. The combination of Indigenous and western research methods helps strengthen the cultural and scientific credibility of findings [35].

**Fig. 1:**
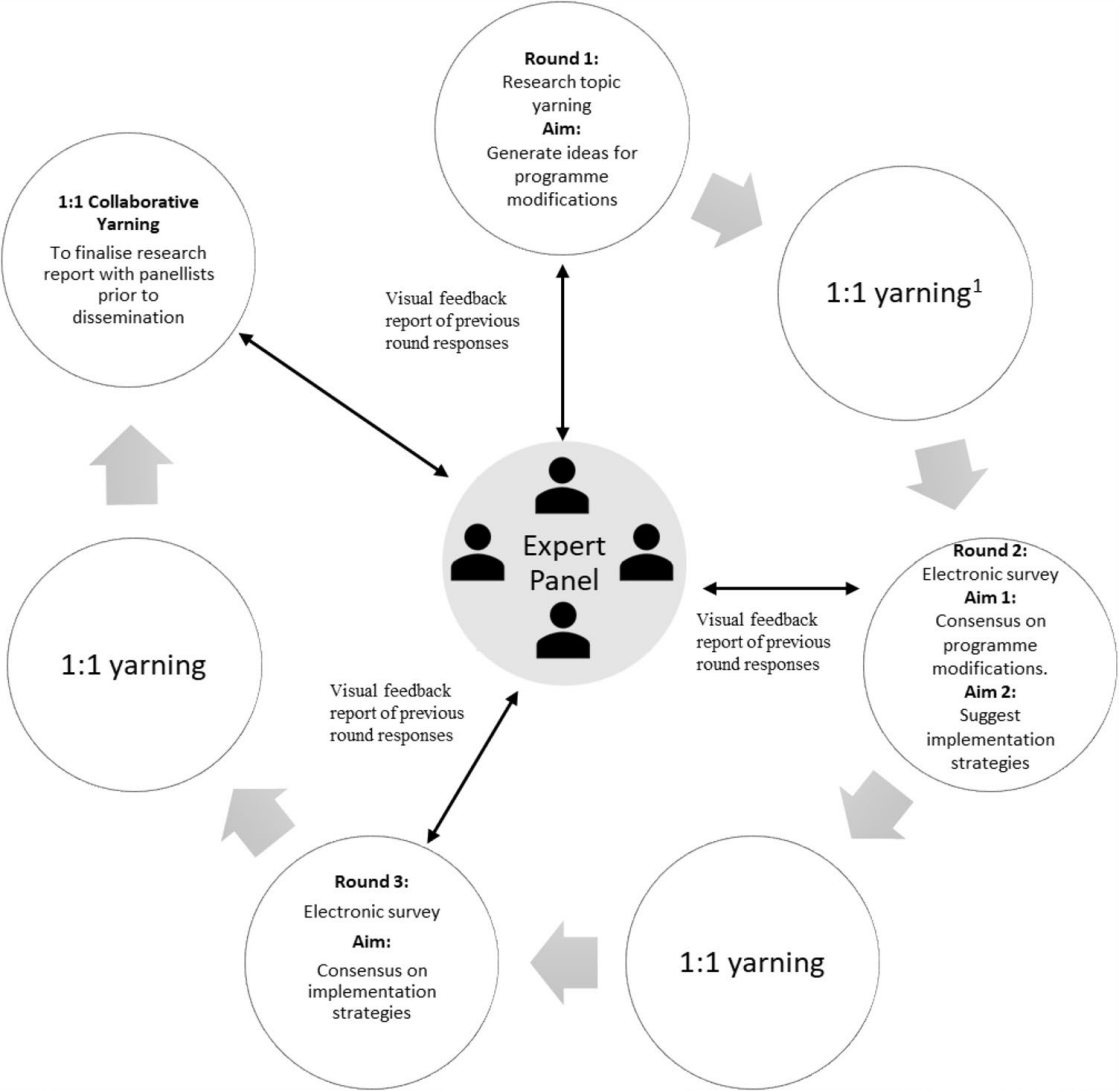
Study design. ^1^ 1:1 Yarning occurred individually between researcher and a panellist

The study design (Fig. 1) adhered to the four fundamental Delphi requirements: anonymity, iteration, controlled feedback, and statistical analysis of group responses [36]. Research topic yarning (conducted 1:1 with each panellist and ED via phone) was incorporated into the design to establish respectful and reciprocal relationships between the researcher and panellists prior to initiating the Delphi process. Yarning was continued (via phone, text and email) in between survey rounds to promote maximum contribution of the expert voice [27]. The decision to conduct three Delphi rounds was made in collaboration with panellists to determine a level of involvement that did not compromise cultural, community or professional obligations or the integrity of the Delphi technique. A similar approach to reduce participant burden was used in a New Zealand study involving both Maori and non-Maori panellists [37]. Three Delphi rounds has been shown to be sufficient to achieve group consensus [38]. Collaborative yarning [27] (yarning purposed to share and explore research ideas) was also conducted after each Delphi round to enable panellists to contribute to study write up.

### Formation of the panel

In the absence of literature confirming an optimal Delphi panel size [39], we sought to recruit panellists with sufficient expertise [28] and within the recommended panel size of 8–12 experts [31, 40].

#### Panel selection criteria

Selection criteria for the panellists were: (1) aged 18+ ; (2) self-identify as being of Aboriginal and/or Torres Strait Islander descent; (3) a minimum of two years of work or academic experience in an Indigenous-specific drug and alcohol, mental health and/or related health and wellbeing field (not necessarily continuous); and (4) basic computer proficiency with reliable access to a computer and internet for the study duration. Panellists were not required to have prior experience with SMART Recovery or other mutual support group programmes. This was because impartiality can strengthen Delphi results [28]. Efforts were made to recruit even numbers of women and men and Indigenous peoples from different community contexts.

#### Panel recruitment

All panellists were recruited using purposive sampling. Panellists were invited to participate by a personalised email or phone call (ED). Four panellists had professional or academic connections with the research team (ED, KL, KCo, JC, RI, KCl and PK). Another six panellists were trained SMART Recovery facilitators who were known to the researchers via other studies. The remaining panellist was recruited via recommendation from another panellist. Anonymity was protected by de-identifying all data and corresponding with panellists individually.

### Ethics and informed consent

Ethical approval was granted by the University of Wollongong (#2018/398), the Aboriginal Health Council of South Australia (#04–19-845), the Western Australian Aboriginal Health Ethics Committee (#939) and the Aboriginal Health and Medical Research Council of New South Wales (#1447/18). All participants provided written and verbal consent through an informed process.

## Procedure

### Data collection

All data were collected between March and July 2020 (by ED). Qualitative and quantitative data were collected across each of the three sequential rounds. Round 1 involved 1:1, telephone-based, research topic yarning (yarns). Rounds two and three used an electronic survey. A portion of panellists (n = 5) provided additional qualitative information in between the Delphi rounds (e.g. justifications for responses and suggestions regarding research implications). These data were aggregated into the accumulating pool of data and analysed accordingly. An a priori consensus level of 80% was set prior to each survey round.

### Round 1: Individual research topic yarns

Individual research topic yarns were conducted (by ED with each panellists) to build rapport and to initiate the Delphi process. Because research topic yarning can either be unstructured or semi-structured [27], all yarns involved an open dialogue to obtain panellists’ freely expressed views and opinions [41]. Yarns also comprised of a series of pre-planned yarning questions to ensure qualitative and quantitative information was systematically collected. Yarning questions were piloted (by ED) with an Aboriginal Elder prior to administrations. All yarns were transcribed using hand-recorded notes (ED). To ensure transcript accuracy, care was taken to record responses verbatim [42] and verbal confirmation was sought from each panellist of the written accounts as the yarns progressed. Three panellists asked to see the yarning script prior to participating in a yarn. Each panellist provided written responses to the script (via email) in addition to participating in a 1:1 phone yarn.

Panellists were asked to prepare for their yarn by reviewing an electronic version of the SMART Recovery Aboriginal and Torres Strait Islander programme handbook (provided to them by ED). The structured yarning questions asked panellists to provide their biographic characteristics (e.g. age, gender, Indigeneity, educational background, professional experience, and level of familiarity with SMART Recovery). Panellists were then asked two quantitative questions: (1) How culturally appropriate is the handbook? and (2) How well do you think the handbook communicates the elements of the SMART Recovery programme for an Indigenous audience? Responses used a ranking scale (0–10).

Yarning was then used to elicit panellists’ impressions of the handbook and to generate a list of modifications (i.e. adaptations, omissions, inclusions) they felt would enhance its cultural utility for Indigenous Australians. A series of prompts sought panellists’ views on culturally appropriate ways to use imagery, language, literacy and programme activities—in relation to programme content, design and delivery. These prompts were drawn from previous research that showed these are areas of mutual support group programmes most commonly modified by Indigenous peoples [23–25].

### Rounds 2 and 3: Electronic surveys

Each survey was pilot tested for accuracy, usability and timeliness prior to dissemination by members of the research team (n = 3) and by Indigenous and non-Indigenous peoples not involved in the study (n = 5).

Both survey rounds were initiated by email to panellists (ED; individually and simultaneously). Each email contained a unique electronic survey link and a visual feedback report detailing the previous round’s group responses. Each survey was available for two weeks. Reminder emails were sent manually after seven days to non-responders.

### Round 2

The aim for Round 2 was to: (1) Achieve group consensus on the list of proposed programme modifications (derived from Round 1); and (2) Solicit suggestions for how each modification could be practically implemented.

Panellists used a 5-point Likert scale to rate 15 proposed programme modifications. They were asked to indicate their level of agreement on each modification’s ability to enhance the cultural utility of the programme (strongly agree through to strongly disagree). Panellists were then given a free-text box to suggest how each modification could be practically implemented. These text boxes also allowed panellists to make other comments as needed.

### Round 3

The aim of Round 3 was to obtain consensus on an accepted set of strategies to enable implementation of the suggested programme modifications. The panellists were presented with a table divided into five key programme modifications. They were asked to either “accept” or “reject” a series of implementation strategies assigned to each. A free-text box was provided for panellists to list reasons why an implementation strategy was rejected. Panellists were also asked to: re-rate two items that did not reach consensus (during Round 2); order their preferences for four proposed handbook titles; and answer four closed questions about this Delphi experience. A free-text box asked for suggestions on how the Delphi technique could be improved for future Indigenous-focused research.

## Data analysis

### Round 1: Individual yarns

Yarning transcripts were prepared for analysis by de-identifying and converting each from handwritten notes into electronic files (Microsoft Word: qualitative data; Excel: quantitative). Qualitative data were analysed manually (ED) using thematic content analysis [28, 31, 43]. This involved an initial open-coding phase of each transcript to identify themes, followed by a focused phase to collapse themes into major categories. All transcripts were checked for coding (KL) and discussed (ED, KL) to reach agreement. Quantitative data were analysed using descriptive statistics.

### Rounds 2 and 3: Electronic surveys

All survey data were collected using REDCap (Research Electronic Data Capture) [44].

## Results

### Panellists

Panellists were 11 Indigenous Australian health and wellbeing experts representing six communities spanning rural, remote and urban contexts (Yuin, Gadigal and Bunjalung—New South Wales, NSW; Nyungar—Western Australia, WA; Nukunka and Kaurna—South Australia, SA). As shown in Table 1, there were six men and five women, with a mean age of just under 50 years (range: 33–65 years). Just over half of the panellists (n = 6/11) were trained SMART Recovery facilitators, and of these, four were facilitating Indigenous-specific SMART Recovery groups. Of the remaining panellists, four had prior knowledge of SMART Recovery (and of AA) via their professional networks. One panellist had no knowledge or experience with any mutual support group programmes.

**Table 1:** Panellists (n = 11) socio-cultural characteristics

Age	54	42	33	50	54	65	53	57	34	34	51
Gender	Female	Female	Female	Female	Female	Male	Male	Male	Male	Male	Male
Indigeneity	Aboriginal	Aboriginal	Aboriginal	Aboriginal	Aboriginal	Aboriginal	Aboriginal	Aboriginal	Aboriginal	Aboriginal	Aboriginal
State	NSW	WA	NSW	SA	WA	NSW	NSW	SA	NSW	SA	NSW
Education background	Bachelor of Nursing	XX/PhD candidate	Bachelor of Nutrition science	Bachelor of Social Work	Post graduate diploma in counselling	Master of Indigenous health/PhD candidate	Grad Dip Indigenous Health	Diploma of Ministry	PhD	Diploma of Counselling	Bachelor of Education/ Diploma of Counselling
Professional background	Nursing; AOD counselling; mental health clinician; Aboriginal health research	AOD harm reduction and prevention; Aboriginal health research	administration in educator sector; public service; Aboriginal health research	social work; AOD counselling	AOD counselling, health service manager	AOD counselling; health service manager Aboriginal health research	AOD counselling; Aboriginal health project development and research	AOD counselling	Education; primary health care service design and evaluation; Aboriginal health research	Social and emotional wellbeing counselling, AOD counselling	Gambling counselling; Aboriginal health research
SMART Recovery connection	Trained facilitator	Knowledge of programme via research projects	No knowledge of programme	Trained facilitator	Works alongside SMART Recovery facilitators and groups	Trained facilitator	Trained facilitator	Trained facilitator	Knowledge of programme through professional connections	Trained facilitator	Is not formally facilitator trained but has co-facilitated groups

*NSW* New South Wales, *WA* Western Australia, *SA* South Australia, *AOD* Alcohol and other drug

#### Education and professional expertise

Panellists had a range of clinical and research expertise that collectively offered more than 190 years of Indigenous health-related work experience. The panellists were working in a variety of settings including a state-funded health service (n = 3), university (n = 3), non-government welfare organisation (n = 1), and an Aboriginal Community-Controlled health service (n = 4). Educational qualifications ranged from diploma level (n = 3) to Doctor of Philosophy (n = 1 completed; n = 2 candidates).

### Delphi rounds

#### Round 1

There was 100% participant retention rate across all three Delphi rounds. The panellists scored the cultural appropriateness of the handbook as 4.3 out of 10 (SD = 2.5). Their rating for how well the handbook communicated the elements of the SMART Recovery programme for an Indigenous Australian context was slightly higher at 5.5 out of 10 (SD = 2.9). Fifteen proposed modifications emerged following thematic analysis of the yarning transcripts (see Table 2).

**Table 2:** Results from Round 2: showing the variation in group agreement for each of the 15 proposed programme modifications (consensus level 80%)

100% Group agreement	
The handbook should be divided into a separate facilitator guide and attendee workbook The handbook(s) have the capacity to use artwork and images representative of different communities The handbook(s) convey a progressive storyline of a person applying SMART Recovery meetings and program tools within their recovery journey The handbook should include cultural symbolism (e.g. Aboriginal and Torres Strait Islander flags) Include activities that incorporate family and community Include activities that promote healthy cultural identities Translate the core SMART Recovery tools and activities using Aboriginal validated and/or designed resources (e.g. The Stages of Change model developed from the NT Living with Alcohol program with artists from Titjikala community – Terry Simmons and Sophia Conway)	

90% Group agreement	
Use strengths-based wording Use gender images respectfully (i.e. be considerate when presenting images of women within a men’s group)	

82% Group agreement	
Rewrite the handbook to accompany varying levels of literacy Use language that is localised to different communities The handbooks should be short	

(72%) Group agreement below consensus level	
The handbook should have the capacity for each community to use locally relevant scenarios as examples of applying SMART tools and techniques^a^ Creating an audio version of the handbook(s) would be useful for some people/communities^b^	

^a^Item was re-rated during round 2 and reached consensus (90%)

^b^Item was re-rated during round 2 and reached consensus (90%)

#### Round 2

During Round 2, almost all of the proposed modifications achieved group consensus (n = 13/15; ≥ 80%). Just over half of the modifications (n = 8/15) achieved perfect consensus. Table 2 shows the different levels of agreement achieved for each of the 15 proposed modifications. Two modifications did not reach consensus (each scoring 72%). These were: “The handbook should have the capacity for each community to use locally relevant scenarios as examples of applying SMART tools and techniques” and “Creating an audio version of the handbook(s) would be useful for some people/communities”.

All panellists used the free-text survey boxes provided to offer suggestions for how the proposed modifications could be practically implemented. Their responses generated an initial list of 80 implementation strategies that were reduced to 29 items by removing duplicates and during thematic content analysis (See Table 3). Themes were checked (by KL) and discussed (ED, KL) to reach consensus. Emerging themes were verified with panellists (n = 6) who utilised a 1:1 yarning opportunity (with ED) in between survey rounds.

**Table 3:**
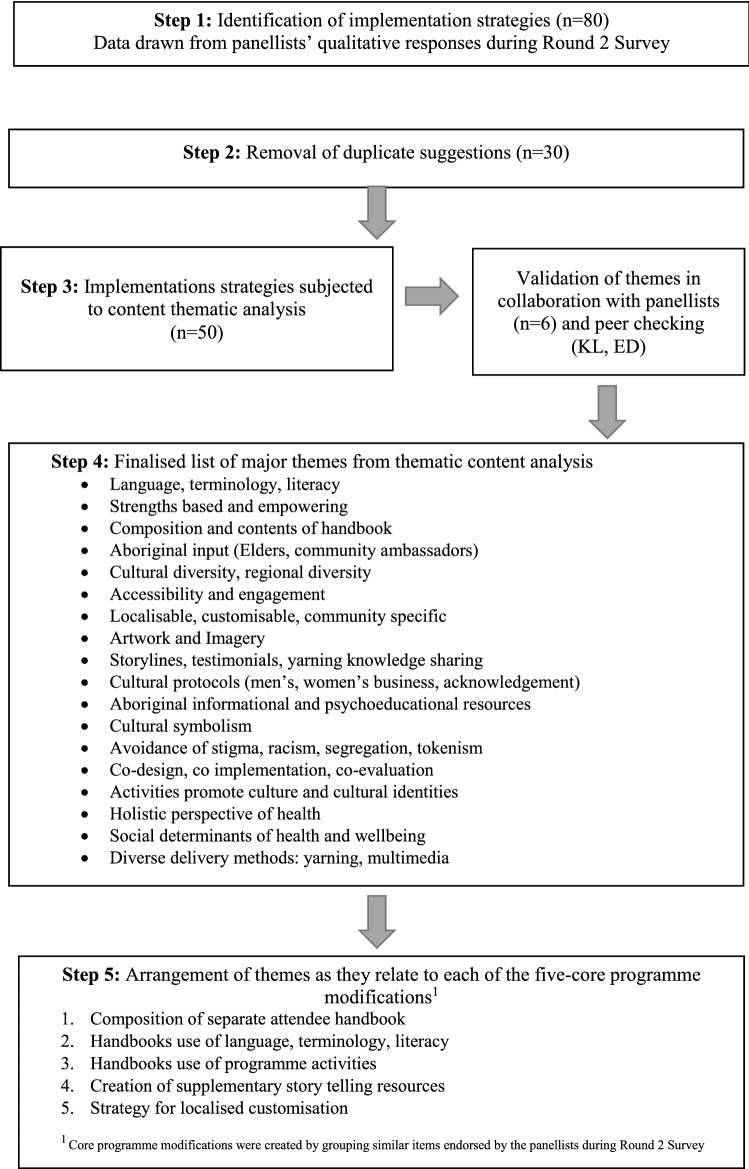
Data analysis approach taken to identify and categorise the panellists list of agreed implementation strategies

Between Rounds 2 and 3, the endorsed list of 13 modifications was refined by grouping similar concepts together. This created five core categories defining key aspects of the programme that the panel recommended be changed (see Table 3). The 29 implementation strategies were then arranged according to the modification they related to.

#### Round 3

In this round, all but one of the 29 implementation strategies were accepted (n = 28/29) (Table 3). The one strategy that was rejected (by n = 4/11) was: to “Prepare handbook(s) as generic templates with no imagery and simple language”. This related to the key modification “strategy for localised customisation”. The panellists’ reasons for rejecting this strategy was the belief that being responsible for customising SMART Recovery programme materials would be a burden on local facilitators. Two alternative strategies were proposed by these four panellists: (1) To create a customised handbook during facilitator training and, (2) For SMART Recovery to [practically and financially] support facilitators to create a handbook on return to their communities after training is completed. The final set of accepted implementation strategies are presented in Table 4.

**Table 4:** Final list of programme modifications and implementation strategies achieved

Implementation strategies accepted after Round 3		
Key programme modification	Accepted strategy	Panellists’ quotes
1. Composition of a separate facilitator and group member handbook	1. Is no more than 15 pages	“Keep it simple, 10–15 pages max”
	2. Has space for writing, drawing, and working through activities	“If it’s a handbook for participants then make it about them. [it needs to be] long enough to convey [the programme] concepts, provide workspace and be short enough to not be overwhelming to use”
	3. Use Indigenous designed and/or developed recovery resources	“Aboriginal validated and designed resources should be used”
	4. Have a minimal amount of written text (higher ratio of artwork and imagery)	“I think it should be easy to read and follow without huge chunks of text”
	5. Convey the core programme tools and techniques using artwork and imagery	“The language is clinical and unfamiliar for many Aboriginal and Torres Strait Islander mob, especially those for who English is a 3rd language. Use pictures to convey ideas where possible and ensure it is written in the right voice and style. Add a glossary for those terms that cannot be substituted to explain meanings
	6. Contains testimonials of Indigenous people who have recovered attending SMART Recovery groups	“[It would be helpful to] share testimonies of facilitators or an Aboriginal person who has [recovered by using] the programme and has moved forward to a point of no longer being an addict, or have an addiction or if so has ways of managing it well with the right supports in place through key Elders etc.”
2. Culturally appropriate language, terminology, and literacy level	7. Reflects the voice of Aboriginal and Torres Strait Islander peoples	“When we talk about terms like 'meetings' or 'program tools' it does not apply to our ontology, terms to need to define in our way of knowing, being and doing”
	8. Is clearly written	“Any piece of writing that is simple and concise will be able to communicate it's intended message across easier”
	9. Is strengths based,	“Strength based wording would hopefully give people a sense of empowerment”
	10. Is empowering	“I would love to see the shame taken out of recovery and empower participants to own their story and their journey wherever they may be on it.”
	11. Is engaging	“The attendee workbook must be written in language that conveys the voice and perspective of ATSI peoples or we won’t engage with it”
3. Culturally meaningful programme activities	12. That can strengthen connections to community and country	“Healing happens at the community level”
	13. That strengthen cultural identities	“[there needs to be] activities where attendees could be culturally immersed and promote their own cultural wellbeing”
	14. That encourage positive social, family and community support networks	“It is proven that Aboriginal people confront and tackle serious issues/problems collectively, the reason for this is so we can add identity, family kinship and culture to everything we do”
	15. Promote holistic concepts of health and wellbeing	“What we were doing was running fitness programmes for the clients as they progress this helps them deal with their cravings and urges etc.”
4. Create supplementary storytelling resources	16. Co-created with a range of different community ambassadors	“I would like to see the [new] handbook be co-created by consumers on how they view the world, which would inform the language that should be used. I find it interesting that we talk about consumer-centred care but when we develop intervention strategies it neglects the voice of the consumer who live their experience and that intervention strategies should be about facilitation of change not a forceful direction of change”
	17. Narratives reflect diverse culture and community groups	“[this would make the handbook more meaningful for group members] because they know that the book has cultural values [and contains] an Aboriginal perspective not a western world mind set”
	18. Map onto the handbooks in such a way that they reinforce learning of tools and techniques (e.g. a story would be created to exemplify how to use urge surfing)	“[It would be more helpful if the handbook used] examples of local programs or people, or even a case study that uses local language and terms”
	19. Promote holistic concepts of health and wellbeing	“This would help participants identify with the content. I don't seem to see this in the current [handbook]”
	20. Address the broader social and historical determinants of recovery, health, and wellness	“because we are constantly being bombarded with negative views of our people”
	21. Complemented by imagery depicting a range of different skin types, genders, and ages	“I suggest a range of gender expression, ages and skin colour. Aboriginality isn’t about colour so we need to stay away from stereotypes full stop”
	22. Depict a progressive journey of how people recover attending SMART Recovery groups	“[the handbook needs] a storyline that conveys a progressive but cultural storyline. [this] cultural storyline would provide attendees the opportunity to nurture their cultural spirituality and their own self-narrative”
5. Customisation for diverse community contexts	23. Provided facilitators with a master shell during or after SMART facilitator training	“[this would allow] the facilitator to reword to suit the audience”
	24. Each facilitator would be responsible for customising the handbook according to their groups context and needs	“The facilitator should be able to reword to suit the audience”
	25. Customisations would include use of local language/terminology (i.e., rugby vs football), local artwork, imagery, and symbolism and, a personalised acknowledgement of Country	“If region specific resources are prepared, language will be easily localised. If language is not localised you risk excluding participants. It [may not always be a matter of] translating into each of the local languages, but maybe using terms and examples that are locally relevant”
	26. Content is tailored to each community's primary substance(s) use and/or problematic behaviour(s) of concern	“This issue is very difficult to achieve, it would be great to design a handbook that relates to each community however trying to accommodate everyone is nearly impossible but would be fantastic”
	27. Customisation includes acknowledgement of Country	“[need to] localize the images and artwork, even the acknowledgment of country where the groups are run should be aligned’

Implementation strategy rejected after Round 3	Panellists alternative suggestions	
Prepare handbooks as generic templates with no artwork or imagery and simple language	1. Create a customised handbook during facilitator training 2. SMART Recovery practical and financial support for facilitators to create a handbook on return to their communities after facilitator training	

Strategies that were added after reaching consensus in Round 3	Participant quotes	
1. Creating an audio version of the handbook(s) would be useful for some people/communities	“Having an audio version would be fantastic and especially if in different Aboriginal languages. People retain and learn information in different ways. Most people [Indigenous or not] need a variety of learning tools”	
2. The handbook should have the capacity for each community to use locally relevant scenarios and symbolisms as examples of applying SMART tools and techniques	“I think it's vital people from different communities can connect with the materials regardless of where they are from”	

The two items from Round 2 that did not reach group consensus were re-rated and both achieved a group consensus (91%). These were then amalgamated into a final set of endorsed programme modifications Panellists preferred the handbook titles: “Stay solid, stay grounded” and “Getting strong and living long” (equal first place); then “SMART Recovery for me and my community” and lastly; “The SMART way to give up”.

Panellists were positive about their experience of being a Delphi study participant:

*“I think the Delphi style was user friendly, clear understanding of what was expected from the participant. Well set out”*

*“It has been a pleasure to be involved”*

*“Look forward to the end product”*

*“Thank you for giving me the opportunity to have my say”*

Almost all panellists (n = 10/11) answered “yes” to: (1) having had enough opportunity to offer their expertise; (2) that their opinions were incorporated and; (3) that they could participate with minimal disruption to their daily work demands. Nine of the eleven panellists felt that a Delphi study was an appropriate method for obtaining Indigenous knowledges. Only one panellist offered a suggestion for how the Delphi method could be improved for future research with Indigenous peoples:

*“Go out to communities and speak with key Elders, get their input, listen to them and expand on what they are trying to achieve”.*

## Discussion

To our knowledge this is the first study to combine an Indigenous research method with the Delphi technique to explore the cultural utility of mutual support group programmes. This study assembled a culturally, geographically, and professionally diverse panel of 11 Indigenous Australian health and wellbeing experts. The panel was tasked with reviewing and commenting on the suitability and helpfulness (i.e. cultural utility) of an Indigenous Australian SMART Recovery programme handbook. Over three Delphi rounds the panel reached consensus on five key programme modifications and developed a set of strategies to help SMART Recovery integrate them into future programme planning and design. These findings offer promise for improving Indigenous Australians’ access to SMART Recovery [45, 46] and is an important first step in determining cultural validity of this programme for Indigenous peoples, globally. They also contribute to creating a more equitable mainstream health care sector [47].

Culture is a critical part of Indigenous people’s health and wellbeing [16, 48]. As such it is vital that internationally available programmes like SMART Recovery consider their cultural utility as this will help to ensure they can meet the recovery needs of Indigenous peoples worldwide [49–51]. Prior to this study, just one other [23] had considered the role of culture within SMART Recovery. Consistent with Dale et al. [23], the current study identified aspects within the model (contents, design and delivery) that, if modified, could improve the programme’s suitability and perceived helpfulness for Indigenous Australians. The need for similar adaptations to improve the cultural utility of Alcoholics Anonymous (AA) has been highlighted by First Nations peoples in the United States of America [21, 25, 52] Common among the endorsed programme modifications were strategies designed to reduce access and engagement barriers. For example, more than a third of implementations strategies (n = 11/29) related to reducing cross-cultural language and literacy barriers [45, 47, 53]. Another seven strategies were focused on how artwork, symbolism, and imagery could make the programme more appealing to a diverse group of Indigenous Australians [54–57].

The panel recommended that the group member handbook be supplemented with storytelling resources and testimonials from recovered Indigenous group members. Storytelling, is a traditional form of therapy used by Indigenous peoples around the world to promote health and healing [58]. Research supports healing narratives (re-storying) as an effective and culturally validated form of treatment for Indigenous [59, 60] and non-Indigenous peoples in recovery from problematic substance use and behavioural addictions [61, 62]. In light of this, SMART Recovery could consider including narrative therapy alongside their current therapeutic approach (of cognitive behaviour therapy and motivational interviewing) [12].

One aspect of the modification process that SMART Recovery may find challenging would be accommodating localised programme customisations [13, 14, 63]. Australia’s Aboriginal and Torres Strait Islander population includes over 250 distinct language and cultural groups [64]. Each community has diverse needs and aspirations [65] and is impacted on uniquely by historical, political and socio-economic determinants of health and wellbeing [66]. As such, the panellists were firm in their recommendation that any future amendments be co-designed, collaboratively implemented and continually co-evaluated via partnerships with representatives from diverse community groups.

All panellists felt that the Delphi technique was a culturally appropriate method to undertake Indigenous-focused research. The Delphi technique has been used in previous studies with Indigenous health and wellbeing professionals from Australia, New Zealand, American and Canada to identify health priorities [67, 68] and develop culturally appropriate treatment guideline and rating scales [37, 69–73]. As methodological adaptations to the Delphi technique are permissible we synthesised Indigenous research methods (collaborative and research topic yarning) [27] alongside the Delphi technique [74]. This was done to maximise contribution of the Indigenous voice, and adhere to the principles of Indigenous research: respect, relationship and reciprocity [32, 33, 75]. This approach is vital to ensure the cultural safety of Indigenous peoples participating in research [76]. It is also an effective way that Indigenous knowledges can be translated into health promoting policies and practices [77].

### Limitations

This study is limited by a small sample size of experts primarily located in New South Wales (n = 6/11). Indigenous voices from regions of Tasmania, Victoria, Northern Territory, Queensland, and the Torres Strait Islands are missing. Likewise, the voices of Indigenous health professionals experienced in non-substance related addictions are would further strengthen the findings. Social desirability bias may have affected some panellists (n = 4) who were known to the research team. However, actions taken to mitigate this included maintaining anonymity between panellists [31], explicit reminders made in each round of data collection that there were no right or wrong answers [78], and by ED positioning herself as a guardian [79] of the Indigenous knowledge holders and knowledges represented within this study.

### Implications

This study contributes to a small but growing body of research showing the need to modify mainstream mutual support groups to be more suitable and helpful for Indigenous Australians. By consulting with Indigenous Australian health and wellbeing professionals, this study makes explicit the areas within the SMART Recovery programme that require cultural modification. A developed set of implementation strategies are offered to help SMART Recovery prioritise areas for change.

Future research is needed to expand our understanding of how the SMART Recovery programme could be most relevant and helpful for Indigenous peoples worldwide. This would require drawing on the knowledges of Indigenous health and wellbeing professionals and Indigenous SMART Recovery facilitators and groups members from more diverse Indigenous communities. It would be important to include Indigenous peoples internationally who have not yet had the chance to provide their perspective of the SMART Recovery programme.

Future research is also needed to determine the cultural utility of other popular mutual support groups programmes (e.g. AA and GA). Once cultural utility has been determined it will be important to culturally validate these programmes to ensure the needs and preferences of all Indigenous peoples (Australian and worldwide) are being supported. The cross-cultural methodology used within this study could assist such work.

This Indigenous-lensed Delphi study appeared to be a culturally appropriate and practical method for conducting Indigenous-focused research. Future studies could consider the role of video conferencing (1:1) which has particular relevance given difficulties engaging in face-to-face data collection due to COVID-19. Video (i.e. face-to-face) rather than phone conferencing, is also more aligned to Indigenous ways of communicating [29], and could help establish trust and rapport between participant and researcher [80].

## Conclusions

This study helps fill important empirical gaps in how to improve the cultural utility of mainstream mutual support groups for Indigenous peoples. The study findings highlight the importance of involving Indigenous peoples in the design, delivery and validation of mainstream mutual support programmes. Programmes that lack Indigenous input can perpetuate biases within mainstream health care approaches and impede Indigenous peoples’ access to equitable and appropriate care.

By embedding Indigenous research methods (yarning) with the Delphi technique, this study offers a culturally appropriate, efficient, and collaborative way that Indigenous cultural knowledges can be integrated into health care policy and practice. It is possible that this approach could help give voice to Indigenous peoples more globally. This study design may also help other mainstream mutual support groups programmes (like AA) evaluate and enhance their cultural utility and validity for Indigenous peoples in similarly colonised countries (i.e. United States of America, Hawaii, Canada and New Zealand).

## Data Availability

Data for this project is stored at the University of Wollongong based at Northfields Avenue Wollongong, New South Wales, 2500 Australia.

## Abbreviations

AA: Alcoholics anonymous
GA: Gamblers anonymous
